# Identification of Germinal Center B Cells in Blood from HIV-infected Drug-naive Individuals in Central Africa 

**DOI:** 10.1080/10446670410001670454

**Published:** 2004-03

**Authors:** Lydie Béniguel, Evelyne Bégaud, Fabrice Cognasse, Philippe Gabrié, Christophe D. Mbolidi, Odile Sabido, Mary A. Marovich, Christiane deFontaine, Anne Frésard, Frédéric Lucht, Christian Genin, Olivier Garraud

**Affiliations:** 1 GIMAP EA3064 Faculté de Médecine Université Jean Monnet 15 Rue A. Paré Saint Etienne cédex 2 42023 France; 2 Institut Pasteur de Bangui Bangui Central African Republic; 3 Hôpital Communautaire Bangui Central African Republic; 4 Centre Commun de Cytométrie en Flux Faculté de Médecine Université Jean Monnet 15 Rue A. Paré Saint Etienne cédex 2 42023 France; 5 Combined US Military HIV research Program Rockville MD USA; 6 Service des Maladies Infectieuses University Hospital Saint Etienne France

## Abstract

To better understand the pathophysiology of B cell populations—the precursors of antibody secreting cells—during chronic human immunodeficiency virus (HIV) infection, we examined the phenotype of circulating B cells in newly diagnosed Africans. We found that all African individuals displayed low levels of naive B cells and of memory-type CD27^+^ B cells, and high levels of differentiated B cells. On the other hand, HIV-infected African patients had a population of germinal center B cells (i.e. CD20^+^, sIgM^-^, sIgD^+^, CD77^+^, CD138^±^), which are generally restricted to lymph nodes and do not circulate unless the lymph node architecture is altered. The first observations could be linked to the tropical environment whereas the presence of germinal center B cells may be attributable to chronic exposure to HIV as it is not observed in HIV-negative African controls and HAART treated HIV-infected Europeans. It may impact the management of HIV infection in countries with limited access to HIV drugs and urges consideration for implementation of therapeutic vaccines.


*Clinical & Developmental Immunology*, March 2004, Vol. 11 (1), pp. 23–27

Taylor & Francis healthsciences

### Identification of Germinal Center B Cells in Blood from HIV-infected Drug-naive Individuals in Central Africa

LYDIE BÉNIGUEL[a], EVELYNE BÉGAUD[b], FABRICE COGNASSE[a], PHILIPPE GABRIÉ[c], CHRISTOPHE D. MBOLIDI[c], ODILE SABIDO[d], MARY A. MAROVICH[e], CHRISTIANE DeFONTAINE[f], ANNE FRÉSARD[f], FRÉDÉRIC LUCHT[f], CHRISTIAN GENIN[a] and OLIVIER GARRAUD[a,*]

[a]*GIMAP EA3064, Faculté de Médecine, Université Jean Monnet, 15 Rue A. Paré 42023, Saint Etienne cédex 2, France;* [b]*Institut Pasteur de Bangui, Bangui, Central African Republic;* [c]*Hôpital Communautaire, Bangui, Central African Republic;* [d]*Centre Commun de Cytométrie en Flux, Faculté de Médecine, Université Jean Monnet, 15 Rue A. Paré 42023, Saint Etienne cédex 2, France;* [e]*Combined US Military HIV research Program, Rockville, MD, USA;* [f]*Service des Maladies Infectieuses, University Hospital, Saint Etienne, France*

To better understand the pathophysiology of B cell populations—the precursors of antibody secreting cells—during chronic human immunodeficiency virus (HIV) infection, we examined the phenotype of circulating B cells in newly diagnosed Africans. We found that all African individuals displayed low levels of naive B cells and of memory-type $CD27^+$ B cells, and high levels of differentiated B cells. On the other hand, HIV-infected African patients had a population of germinal center B cells (i.e. $CD20^+$, $sIgM^-$, $sIgD^+$, $CD77^+$, $CD138^{\pm}$), which are generally restricted to lymph nodes and do not circulate unless the lymph node architecture is altered. The first observations could be linked to the tropical environment whereas the presence of germinal center B cells may be attributable to chronic exposure to HIV as it is not observed in HIV-negative African controls and HAART treated HIV-infected Europeans. It may impact the management of HIV infection in countries with limited access to HIV drugs and urges consideration for implementation of therapeutic vaccines.

*Keywords*: HIV; B cells; Germinal center; Blood; Lymph nodes

*Abbreviations*: LN, lymph nodes; GC, germinal center; VL, viral load; CAR, Central African Republic

### INTRODUCTION

The human immunodeficiency virus (HIV) induces profound disturbances in cellular and humoral immunity, both at the specific and non-specific levels (Fauci *et al.*, 1996). Typically, untreated HIV-infected individuals experience disease progression and manifest hypergammaglobulinemia with antibodies (Abs) to core and surface components of HIV, but lack specific neutralizing anti-HIV envelop Abs and specific recall Abs to naturally encountered or vaccine delivered antigens (Ag) (Amadori and Chieco-Bianchi, 1990). A classic feature of HIV-disease progression is the consistent destruction of lymph nodes (LN) contributing to the progressive loss of the $CD4^+$ T-cell compartment—the hallmark of HIV infection and AIDS (Richard *et al.*, 2002).

Management of HIV-infected individuals in Africa—and particularly in poor countries—represents a real challenge. Few strategies are in place in tropical Africa, and while the introduction of antiretroviral drugs is planned, only vaccination strategies can truly represent a chance to limit infection spreading (Marcus *et al.*, 2002). Whether these vaccines are expected to be preventive or therapeutic, their ultimate goal is to limit viral replication to favor the expansion of specific cytolytic T cells (CTLs) and induce B cells to produce neutralizing Abs (Peters, 2001). These strategies may be reinforced with the help of an intact innate immune system (Siegal and Spear, 2001).

In the present study, we characterized the peripheral B cell compartment of 15 drug-naive HIV-infected individuals. At homeostasis or in disease states that do not affect LN architecture or bone marrow production, blood B cells are comprised of naive and non-naive (mostly memory) B lymphocytes which recirculate, especially after antigenic exposure. These B cells represent a milestone of the specific infection they defend against as they differentiate into (neutralizing) Ab-secreting cells and, therefore are targets of vaccination strategies. Indeed, the presence of circulating cells assumed to be plasmocytes—capable of secreting anti-HIV Abs—has been reported in $HIV^+$ patients; these studies examined treated Americans or untreated, Europeans with low viral load, respectively (Marcus *et al.*, 2002; Richard *et al.*, 2002). In the present work, we studied a group of HIV-infected individuals

*Corresponding author. Tel.: +133-4-77-42-14-67. Fax: +133-4-42-14-86. E-mail: olivier.garraud@univ-st-etienne.fr

ISSN 1740-2522 print/ISSN 1740-2530 online © 2004 Taylor & Francis Ltd
DOI: 10.1080/10446670410001670454

living in Central Africa, and unexpectedly observed germinal center (GC) originating-B lymphocytes (not plasmocytes) circulating in the periphery. This observation may impact future vaccination strategies.

### MATERIALS AND METHODS

### Patients

African donors were consultants at the "Hôpital Communautaire" of Bangui, Central African Republic (CAR) as spouses of hospitalized patients with AIDS. The prevalence of HIV-infection in CAR is estimated to be 12.9% of the adult population (UNAIDS/WHO 2002). All donors enrolled in this study were informed and consented to donate blood for research purposes, according to the rules of the *ad hoc* Ethics Committee set up by the Institut Pasteur in Bangui, in the absence of a National Ethics Committee. None of the blood donors were previously diagnosed with HIV infection, and all were indeed antiretroviral drug-naive. Patients were selected on the basis of African AIDS symptoms defined by the WHO at Bangui in 1985 (Diagnosing symptomatic HIV infection and AIDS in adults, 1993). Approximately 10 ml of heparinized blood was obtained from a series of individuals and 15 were diagnosed $HIV^+$. HIV serology was routinely followed in Bangui using the Vironostika HIV-Uniform II plus O test (Organon Teknika, Durham, NC). Plasma viral load (VL), determined in Saint-Etienne using a nested PCR technique as previously described (Peters, 2001), ranged from 10,000 to 80,000 copies/ml. HIV-1 clades A predominate in Bangui (Muller-Trutwin *et al.*, 1999). Matched controls consisted of 9 HIV-negative patients African PBMC samples.

In order to compare certain biological parameters, the phenotype of blood B cells from European volunteer blood donors was also tested in this survey. Informed consent was obtained from the European patients according to the requirements of the National French Ethics. There were 30 $HIV^+$ European blood donors followed by the Department of Infectious Diseases at the University Hospital in Saint Etienne, France. Approximately 10 ml of heparinized blood was obtained specifically for the present study. All patients enrolled in this study were treated with antiretroviral drugs according to current protocols and were monitored for clinical and biological parameters for disease progression. Plasma VL was routinely monitored in patients over time. At the time of sampling for the present work, 28/30 patients had VL of less than 50 copies/ml. Matched controls consisted of 9 HIV-negative patients European PBMC samples.

### T and B Cell Phenotyping

Standard T ($CD4^+$) cell phenotyping was performed extemporarily at the Institut Pasteur in Bangui. Lymphocyte subsets were analyzed on a FACSCalibur flow cytometer (Becton Dickinson Immunocytometry Systems, San Jose, CA) with two monoclonal antibodies (MAbs) (aCD4–aCD8, Becton Dickinson Immunocytometry Systems).

The extended B cell phenotyping was performed in Saint-Etienne. PBMCs, stored in Stabilcyte™ (BioErgonomics, St Paul, MN) in Africa, were stained with the following mAbs conjugated with fluorescein isothiocyanate (FITC), phycoerythrin (PE) or RPE-Cy5 suitable for three-color flow cytometry. The following markers were used: CD20-RPECy5, IgD-FITC, IgM-PE, CD38-FITC, CD38-PE, CD27-PE (all from DAKO, Copenhaguen, Denmark); CD77-FITC (BD-Pharmingen, San Diego, CA); CD138-PE (Coulter-Immunotech, Marseille, France). Ig isotype-matched FITC-, PE- and RPECy5-conjugated mouse Ab (DAKO) were used as negative controls to test for non-specific staining. Briefly, $0.5 \times 10^6$ cells were stained with the appropriate mAb for 45 min on ice. Afterwards, the cells were washed in PBS with 10% fetal calf serum and fixed in PBS containing 4% paraformaldehyde. Flow cytometry was performed on the following day using a FacsVantage-SF BD and the CellQuest-Pro™ software (Becton-Dickinson, San-Jose, CA).

### Statistics

Means ± SEM of data between the blood donor groups were compared using the Mann–Whitney test. Statistical analysis was performed using the Statview™ software (SAS Institute INC, Cary, NJ).

### RESULTS

### African Blood Donor Group

The design of the present survey, i.e. the study of blood B cell of volunteer, HIV-infected African individuals unaware of their infection, living with AIDS-spouses, has considerably limited the size of this cohort. The initial serological test performed on blood samples for each volunteer defined two groups ($HIV^+$ and $HIV^-$). Newly recognized $HIV^+$ individuals were informed of their serological status, counseled by the physician at the hospital and entered into the specific HIV program at the clinics. The mean $CD4^+$ T cell count ± SEM was 316.6 ± 65.6 cells/μl for the 15 $HIV^+$ individuals and 913 ± 66 cells/μl for the 11 $HIV^-$ individuals; $P = 0.0007$) (Table I). As very few of the recently diagnosed patients were hospitalized (except #6 and #8) for various reasons (including economical), we were unable to perform CDC staging.

### Major Characteristic of Circulating B Cells in HIV-infected African Blood Donors

The $HIV^+$ African blood donors presented lower B cell counts than the $HIV^-$ Africans. The % mean of $CD20^+$ B

TABLE I Major characteristics of the $HIV^+$ African patients enrolled in the study

| | | Age | CD4 | VL | B cells |
|---|---|---|---|---|---|
| 1 | M | 38 | 163 | 5.5 | 7 |
| 2 | M | 19 | 510 | 4 | 5.5 |
| 3 | M | 43 | 123 | 5.9 | 3 |
| 4 | F | 25 | ND | ND | 5 |
| 5 | M | 39 | 456 | 5.4 | 3.2 |
| 6 | F | 28 | 128 | 5.9 | 4.2 |
| 7 | F | 25 | 956 | 4.26 | 2 |
| 8 | M | 47 | 5 | 5.1 | 2 |
| 9 | M | 22 | ND | ND | 9 |
| 10 | M | 34 | ND | 4.46 | 7 |
| 11 | M | 52 | 13 | 5 | 1 |
| 12 | F | 45 | 181 | ND | 6 |
| 13 | F | 31 | 203 | 4.15 | 3 |
| 14 | F | 32 | 745 | 4.04 | 7 |
| 15 | F | 30 | ND | 5.2 | 9 |

*M* and *F* stand for Male and Female; Age is expressed in years; CD4 count is expressed in $CD4^+$ cells/µl; plasma viral load is expressed in log (copies/ml); B cell is the % of $CD20^+$ cells within PBMC. ND stands for Not determined.

cell count ± SEM was 4.9 ± 0.7 for the 15 $HIV^+$ individuals and 6.7 ± 0.6 for the 9 $HIV^-$ individuals; $P = 0.1$.

A careful examination of the B cell subpopulation phenotype was then carried out by 3 color-flow cytometry. We first determined the relative proportions of naive and non-naive circulating blood B cells on the basis of their reactivity with anti-CD20 and anti-IgM ± anti-IgD fluorescent-dye labeled Abs. As shown in Fig. 1, $CD20^+$, surface-(s)$IgM^+$/$sIgD^+$ cells assumed to be naive B cells (Banchereau and Rousset, 1992) were nearly equivalent in $HIV^+$ than in $HIV^-$ blood donors (28 ± 4.6 vs. 20.4 ± 4.3%, respectively). The $HIV^+$ blood donors also displayed approximately equal $CD20^+$, $sIgM^{\pm}$, $sIgD^-$ differentiated B cells (61.1 ± 4.6 vs. 67.1 ± 7.9%, respectively). In contrast, $CD20^+$ $sIgD^+$ $sIgM^-$ germinal center (GC)-type B cells were significantly higher in $HIV^+$ compared to $HIV^-$ patients (11.9 ± 2 vs. 5.2 ± 0.6%; $P = 0.03$). In total, 8/15 (53%) African $HIV^+$ donors displayed ≥10% of GC-type $CD20^+$ B cells.

To further document these findings, and to ascribe potential functions to the non-naive blood B cells in $HIV^+$ donors, we tested the $CD20^+$ B cells for reactivity with mAbs to CD27 (characterizing circulating memory-type blood B cells), CD77 (characterizing GC-type B cells), CD138 (characterizing post-GC B cells) and anti-CD38 (characterizing pre-plasmocytes and plasmocytes, and certain activated B cells). African $HIV^+$ blood donors displayed similar levels of $CD20^+$ $CD27^+$ memory-type blood B cells compared with their non-infected counterparts (Fig. 2A). However, there were significantly more $CD20^+$ $CD77^+$ GC-type B cells (9.7 ± 1.8 vs. 3.2 ± 0.7%; $P = 0.006$) compared to $HIV^-$ Africans.

Of note, although this cannot be compared *stricto sensu*, unexpected proportions of $CD20^+$ $CD138^+$ post-GC B cells were found in $HIV^+$ and $HIV^-$ Africans compared to as in Europeans (Fig. 2A,B). Moreover, $HIV^+$ and $HIV^-$ African donors had fewer $CD20^+$, $sIgD^+$, $sIgM^+$ naive B cells (28.1 ± 4.6 and 20.4 ± 4.3%, respectively) compared to $HIV^+$ and $HIV^-$ European individuals (72.2 ± 3 and 73 ± 2.2%, respectively). African donors also had more $CD20^+$, $sIgM^{\pm}$, $sIgD^-$ differentiated B cells (61.1 ± 4.6 vs. 23.5 ± 1.9%) and considerably more $CD20^+$ $sIgD^+$ $sIgM^-$ CG-type B cells compared to European patients ($HIV^+$: 11.9 ± 2 vs. 3.2 ± 0.7; $HIV^-$: 5.2 ± 0.6 vs. 3.2 ± 0.7, respectively). Moreover, HIV-infected or uninfected, all African individuals

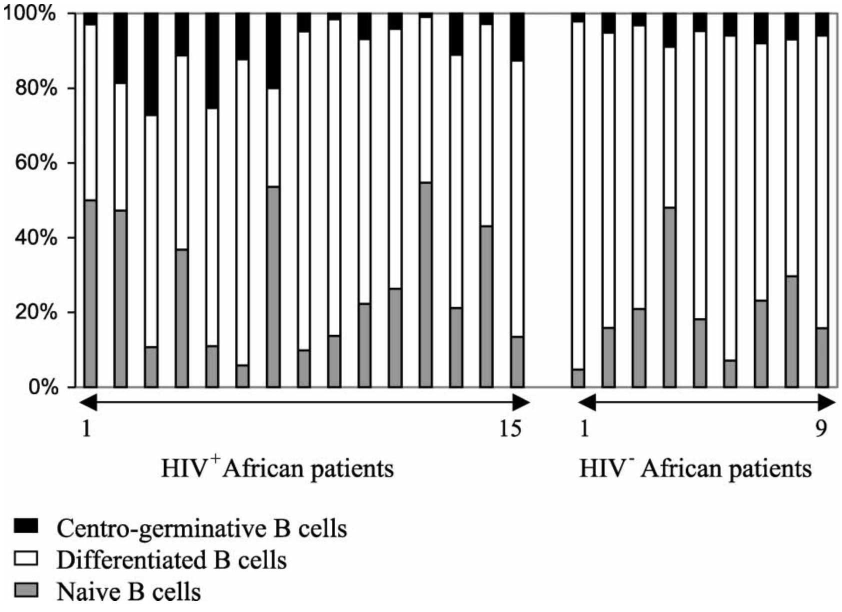

FIGURE 1 Blood B cell phenotype in $HIV^+$ African donors. The bar diagrams show the differential proportion of naive, differentiated and centro-germinative B cells in the periphery (i.e. blood circulating) of HIV-infected, untreated Africans. $CD20^+$, $sIgM^+$ $sIgD^+$ cells are naive B cells (gray bars); $CD20^+$ $sIgM^{\pm}$, $sIgD^-$ cells are differentiated B cells (white bars); and $CD20^+$, $sIgM^-$ $sIgD^+$ cells are centro-germinative B cells (black bars). The analysis shows data obtained with blood from 15 HIV-1 infected patients and 9 HIV-negative patients.

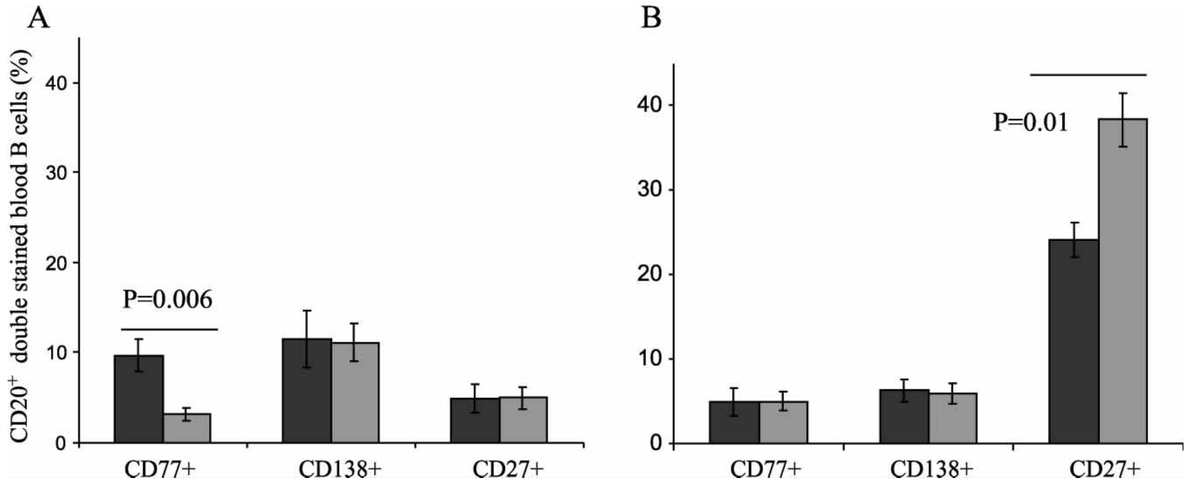

FIGURE 2 Differential expression of CD77, CD138 and CD27 markers on circulating $CD20^+$ B cells. (A) African blood donors; (B) European blood donors; Black bars, HIV-infected individuals; Gray bars, HIV-uninfected individuals.

displayed at least five fold fewer memory-type $CD27^+$ B cells (4.9–5 vs. 24.1–38.4%) than Europeans while $HIV^+$ infected Europeans had significantly less $CD20^+$ $CD27^+$ memory-type B blood B cells than their non-infected counterparts ($P = 0.01$; Fig. 2B).

### DISCUSSION

In this study, we attempted to integrate observations on circulating B lymphocytes in HIV-infected drug-naive individuals. We tried to understand to what extent, and how, the B cell compartment, is affected by HIV-infection, especially in drug-naive patients which represent the majority of infected individuals worldwide. The next step would be to try to circumvent the B cell disorders and in so doing protect the B cell compartment to allow the development of protective-type Abs, awaiting an anti-disease vaccine.

The study population consisted of African individuals unique in four areas: (i) origin and genetic background; (ii) hyperactivation of the immune system by numerous infectious pathogens leading to the so-called tropical AIDS (Germani *et al.*, 1998; Bentwich *et al.*, 2000; Begaud *et al.*, 2003); (iii) no access to antiretroviral treatment and insufficient access to health care and (iv) virus clade.

This and other studies have shown that HIV infection leads to profound alterations at the level of B cell homeostasis (Richard *et al.*, 2002). Although exacerbated plasmacytosis in bone marrow and in peripheral tissues have been consistently reported in HIV infection (Calenda and Chermann, 1992; Nagase *et al.*, 2001), no consensus has been reached on whether plasmocytes can circulate. We did not find circulating plasmocytes in our close examination of blood smears (data not shown). However, we found a fraction of circulating B lymphocytes phenotypically modified when compared to HIV-uninfected individuals, such as the reduction in $CD27^+$ memory-type B cells (De Milito *et al.*, 2001). Certain studies aimed at characterizing the Ab secreting cell precursors found various phenotypic changes such as the low expression of CD21 (Moir *et al.*, 2001), CD23 (Rodriguez *et al.*, 1996), CD38, CD39, CD20, CD37, CD71 (Fournier *et al.*, 2002) and modified expression of CD22, sIgM and sIgG molecules (Dawood *et al.*, 1998) etc, suggesting that these cells could be early plasma cells originating from GCs (Forster *et al.*, 1997). Our study showed that African individuals had fewer naive ($CD20^+$/$sIgM^+$/$sIgD^+$) and memory-type ($CD20^+$/$CD27^+ \pm sIgD^- \ sIgM^{\pm}$) B cells compared to European individuals. This could be linked to the tropical environment where, for example, chronic parasite infections are known to affect the immune system. Multiparasitisme is indeed frequently observed in CAR (Begaud *et al.*, 2003). On the other hand, we found significantly more $CD20^+$/$CD77^+$ B cells (CD77 being a phenotypic characteristic of centroblasts (Wiels, 2000)) in $HIV^+$ Africans compared to HIV-uninfected individuals or European patients. This second particularity seems to be directly associated with the evolution of HIV infection in African individuals and with the presence of the virus (high VL) in the absence of treatment. We also found elevated numbers of B cells expressing surface CD138 (a feature of post-GC B cells) (Wijdenes *et al.*, 1996). However, CD138 marker is imprecisely known, rendering this staining difficult to interpret. Taken together, our findings strongly suggest that the unexpected circulating B cells share the features of GC- (Bm3σ and Bm3) and post-GC-B cells (Liu *et al.*, 1996). *In vivo*, such lymphocytes may have received progression and differentiation signals and/or may be pre-switched. They may differentiate terminally in bone marrow and in tissue-associated lymphoid organs, and some may possibly be aberrantly located as a result of HIV-infection (Forster *et al.*, 1997). It is not clear, however, that these GC cell subsets differentiate *in vivo* and *in vitro* (our unpublished observations) as we have not yet specifically identified a population ascribed to a centrocytic phenotype.

Our data agrees with several studies on the architectural disturbance of the LN in HIV-infection, AIDS and HIV-associated Hodgkin and non-Hodgkin lymphomas (Clarke and Glaser, 2001). Further, it has been reported that disruption of GCs blocks the formation of B cell memory (Roy *et al.*, 2002) which fits with the present observation. Our study may also suggest that the hypergammaglobulinemia observed during HIV-infection at least partly reflects the disorganization of LNs and particularly GCs, which may prematurely release into the periphery GC- and post-GC-B lymphocytes poised to differentiate terminally. Our study differs from other studies in that we have enrolled African patients who were heavily infected with HIV. Our observations focused on the direct relationship between peripheral B cell subset imbalances and LN disorganization, and the absence of treatment (and the absence of viral control). The architectural disorganization of the LN—along with the aberrant release of early plamocytes by the bone marrow—seems largely responsible for the B cell dysregulation observed during HIV-infection progression in African countries. This strongly argues for the establishment of therapeutic vaccines trials as we await the implementation of drug treatment programs.

**Acknowledgements**

We are greatly indebted to the patients who accepted to be enrolled in the study, and to the nurses and medical staff members who contributed to the clinical aspect of this study, particularly to Dr P. Minssart (Hôpital Communautaire, Bangui); Ms J. Leal (Institut Pasteur, Bangui); Dr C. Cazorla, Ms A-M. Lantner and Ms V. Ronat (CHU de Saint Etienne). We also deeply thank Prof. B. Pozzetto, Dr T. Bourlet, Dr O. Delézay, Ms F. Duplat, Mr. D. Laurent and Mr. W. Berlier (Université de Saint Etienne) for their kind help in managing data. We also wish to express our gratitude to Professors J.-L. Durosoir, Y. Buisson and F. Barré-Sinoussi (Institut Pasteur, Paris, and Réseau International des Instituts Pasteur et Instituts Associés); the Fondation Jeunesse Internationale/Fondation de France (Paris); and Dr A. Talarmin (Institut Pasteur, Bangui) for their invaluable support.

### References

Amadori, A. and Chieco-Bianchi, L. (1990) "B-cell activation and HIV-1 infection: deeds and misdeeds", *Immunol. Today* **11**, 374–379.

Banchereau, J. and Rousset, F. (1992) "Human B lymphocytes: phenotype, proliferation, and differentiation", *Adv. Immunol.* **52**, 125–262.

Begaud, E., Feindirongai, G., Versmisse, P., *et al.* (2003) "Broad spectrum of coreceptor usage and rapid disease progression in HIV-1-infected individuals from Central African Republic", *AIDS Res. Hum. Retrovir.* **19**, 551–560.

Bentwich, Z., Maartens, G., Torten, D., Lal, A.A. and Lal, R.B. (2000) "Concurrent infections and HIV pathogenesis", *AIDS* **14**, 2071–2081.

Calenda, V. and Chermann, J.C. (1992) "The effects of HIV on hematopoiesis", *Eur. J. Haematol.* **48**, 181–186.

Clarke, C.A. and Glaser, S.L. (2001) "Epidemiologic trends in HIV-associated lymphomas", *Curr. Opin. Oncol.* **13**, 354–359.

Dawood, M.R., Conway, B., Patenaude, P., *et al.* (1998) "Association of phenotypic changes in B cell lymphocytes and plasma viral load in human immunodeficiency virus-infected patients", *J. Clin. Immunol.* **18**, 235–240.

De Milito, A., Morch, C., Sonnerborg, A. and Chiodi, F. (2001) "Loss of memory (CD27) B lymphocytes in HIV-1 infection", *AIDS* **15**, 957–964.

"Diagnosing symptomatic HIV infection and AIDS in adults". *AIDS Action*, (1993) 6–8.

Fauci, A.S., Pantaleo, G., Stanley, S. and Weissman, D. (1996) "Immunopathogenic mechanisms of HIV infection", *Ann. Intern. Med.* **124**, 654–663.

Forster, R., Schweigard, G., Johann, S., *et al.* (1997) "Abnormal expression of the B-cell homing chemokine receptor BLR1 during the progression of acquired immunodeficiency syndrome", *Blood* **90**, 520–525.

Fournier, A.M., Fondere, J.M., Alix-Panabieres, C., *et al.* (2002) "Spontaneous secretion of immunoglobulins and anti-HIV-1 antibodies by *in vivo* activated B lymphocytes from HIV-1-infected subjects: monocyte and natural killer cell requirement for *in vitro* terminal differentiation into plasma cells", *Clin. Immunol.* **103**, 98–109.

Germani, Y., Minssart, P., Vohito, M., *et al.* (1998) "Etiologies of acute, persistent, and dysenteric diarrheas in adults in Bangui, Central African Republic, in relation to human immunodeficiency virus serostatus", *Am. J. Trop. Med. Hyg.* **59**, 1008–1014.

Liu, Y.J., Arpin, C., de Bouteiller, O., *et al.* (1996) "Sequential triggering of apoptosis, somatic mutation and isotype switch during germinal center development", *Semin. Immunol.* **8**, 169–177.

Marcus, U., Dittmar, M.T. and Krausslich, H.G. (2002) "HIV: epidemiology and strategies for therapy and vaccination", *Intervirology* **45**, 260–266.

Moir, S., Malaspina, A., Ogwaro, K.M., *et al.* (2001) "HIV-1 induces phenotypic and functional perturbations of B cells in chronically infected individuals", *Proc. Natl Acad. Sci. USA* **98**, 10362–10367.

Muller-Trutwin, M.C., Chaix, M.L., Letourneur, F., *et al.* (1999) "Increase of HIV-1 subtype A in Central African Republic", *J. Acquir. Immune Defic. Syndr.* **21**, 164–171.

Nagase, H., Agematsu, K., Kitano, K., *et al.* (2001) "Mechanism of hypergammaglobulinemia by HIV infection: circulating memory B-cell reduction with plasmacytosis", *Clin. Immunol.* **100**, 250–259.

Peters, B.S. (2001) "The basis for HIV immunotherapeutic vaccines", *Vaccine* **20**, 688–705.

Richard, Y., Lefevre, E. and Krzysiek, R. (2002) "B cells in the line of sight of HIV-1", In: Cossarizza, A. and Kaplan, D., eds, Cellular Aspects of HIV Infection (Wiley-Liss, New York), pp 69–102.

Rodriguez, C., Thomas, J.K., O'Rourke, S., Stiehm, E.R. and Plaeger, S. (1996) "HIV disease in children is associated with a selective decrease in CD23 + and CD62L + B cells", *Clin. Immunol. Immunopathol.* **81**, 191–199.

Roy, M.P., Kim, C.H. and Butcher, E.C. (2002) "Cytokine control of memory B cell homing machinery", *J. Immunol.* **169**, 1676–1682.

Siegal, F.P. and Spear, G.T. (2001) "Innate immunity and HIV", *AIDS* **15**, S127–S137.

Wiels, J. (2000) "CD77", *J. Biol. Regul. Homeost. Agents* **14**, 288–289.

Wijdenes, J., Vooijs, W.C., Clement, C., *et al.* (1996) "A plasmocyte selective monoclonal antibody (B-B4) recognizes syndecan-1", *Br. J. Haematol.* **94**, 318–323.

